# Supplementary material for: Life Cycle Transitions in the Freshwater Jellyfish Craspedacusta sowerbii
Source: Biology (Basel). 2024 Dec 20;13(12):1069. doi: 10.3390/biology13121069 (PMC11673735; doi:10.3390/biology13121069)
Supplement: Supplementary file 1 [file biology-13-01069-s001.zip › biology-3129790-supplementary.pdf]

Supplemental Table 1. 16S sequences used for Phylogenetic Reconstruction

GenBank Accession #	Location	Species
AY512507.1	-	*C. sinensis*
EU293974.1	-	*C. ziguiensis*
EU293971.1	Pennsylvania	*C. sowerbii*
JN593332.1	Wuhan, China	
KY077294.1	Unknown	
LN901194.1	Louisville, KY, USA	
MF000530.1	Geneva, Switzerland	
MK600506.1	Morocco	
MK600507.1	Morocco	
MT107153.1	Italy	
MT107154.1	Italy	
MT107155.1	Italy	
MW603820.1	British Columbia, Canada	
MZ146922.1	China	
MZ326744.1	Japan	
MZ508273.1	Europe	
MZ508274.1	Europe	
MZ508275.1	Europe	
MZ508276.1	Europe	
MZ569027.1	unknown	
MZ569028.1	unknown	
MZ569029.1	unknown	
MZ569030.1	unknown	
MZ569031.1	unknown	
NC018537.1	Wuhan, China	
OK037602.1	Japan	
OK037603.1	Japan	
OK037604.1	Japan	
OK037610.1	Japan	
PP954957	Panama (*C.sowerbii*_PAN)	
PP954953	Japan (*C. sowerbii*_JPN)	
PP954956	Lawrence, Kansas (*C. sowerbii*_LKS)	
PP954955	Coal City, Illinois (*C. sowerbii*_CCI)	
PP954954	Inverness, Illinois (*C sowerbii*_INI)	

Supplemental Table 2. P-values for Tukey-Kramer post-hoc tests for the average number of cells in podocysts in three treatments (cold/room temp./ dry) as shown in Figure 4B.

	Cold	Room Temp.	Dry
Cold		0.024*	0.000*
Room Temp.	0.024*		0.152
Dry	0.000*	0.152	

*Significant values ($p < 0.05$)

Supplemental Table 3. Mean number of polyps at day 21 of experiment at different temperatures. Shown are the corrected data for the four strains (see Figure 5).

	Temperature	# of Polyps (mean)	Standard Error
*C. sowerbii*_INI	14°C	10.4	1.18
	22°C	6.9	3.45
	26°C	35.1	12.38
	28°C	26.7	7.07
*C. sowerbii*_LKS	14°C	4.5	0.33
	22°C	14.0	0.55
	26°C	20.0	2.67
	28°C	12.0	3.61
*C. sowerbii*_JPN	14°C	2.7	0.89
	22°C	6.7	3.53
	26°C	9.8	2.85
	28°C	2.3	1.33
*C. sowerbii*_PAN	14°C	10.2*	1.11*
	22°C	12.0*	2.11*
	26°C	13.1*/13.5	0.59*/1.23
	28°C	11.6*/10.4	2.43*/1.20

For *C. sowerbii*_PAN, no data was available at day 21 for the 14°C and 22°C experiments. Thus, for equivalent comparisons, the last data taken at the same time period (between days 17-18) for all temperatures are shown and denoted with an *. Data not denoted with an * was taken at day 21.

Supplemental Table 4. P-values for Tukey-Kramer post-hoc tests within strains for the average number of polyps produced at different temperatures (see Figure 5).

	*C. sowerbii*_JPN				*C. sowerbii*_LKS			
	14°C	22°C	26°C	28°C	14°C	22°C	26°C	28°C
14°C		0.41	0.01*	0.62		0.03*	0.00*	0.09
22°C	0.41		0.04*	0.97	0.03*		0.05*	0.81
26°C	0.01*	0.04*		0.03*	0.00*	0.05*		.018
28°C	0.62	0.97	0.03*		0.09	0.81	0.18	
	*C. sowerbii*_PAN				*C. sowerbii*_INI			
14°C		1.00	0.18	0.87		0.77	0.30	0.63
22°C	1.00		0.19	0.88	0.77		0.08	0.20
26°C	0.18	0.19		0.47	0.30	0.08		0.90
28°C	0.87	0.88	0.47		0.62	0.20	0.90	

*Significant values ($p < 0.05$)

Supplemental Table 5. Comparison of the mean number of polyps per colony at different temperatures (see Figure 6). Shown are P-values for Tukey-Kramer post-hoc tests among means within strains.

	*C. sowerbii*_JPN				*C. sowerbii*_LKS			
	14°C	22°C	26°C	28°C	14°C	22°C	26°C	28°C
14°C		0.74	0.48	0.99		0.13	0.04*	0.12
22°C	0.74		0.13	0.97	0.13		0.83	1.00
26°C	0.48	0.13		0.36	0.04*	0.83		.084
28°C	0.99	.97	0.36		0.12	1.00	0.84	
	*C. sowerbii*_PAN				*C. sowerbii*_INI			
14°C		0.93	0.19	0.19		0.32	0.79	0.89
22°C	0.93		0.41	0.41	0.32		0.79	0.67
26°C	0.19	0.41		1.00	0.79	0.79		0.99
28°C	0.19	0.41	1.00		0.89	0.67	0.99	

*Significant values ($p < 0.05$)
